# The symmetry in the model of two coupled Kerr oscillators leads to simultaneous multi-photon transitions

**DOI:** 10.1038/s41598-023-30197-8

**Published:** 2023-02-21

**Authors:** Bogdan Y. Nikitchuk, Evgeny V. Anikin, Natalya S. Maslova, Nikolay A. Gippius

**Affiliations:** 1 Skolkovo Institute of Science and Technology, 121205 Moscow, Russia; 2 Moscow Institute of Physics and Technology, Dolgoprudny, 141701 Moscow Region, Russia; 3 Russian Quantum Center, 121205 Moscow, Russia; 4 Quantum Technology Centrum, Department of Physics, Lomonosov Moscow State University, 119991 Moscow, Russia

**Keywords:** Quantum physics, Theoretical physics, Atomic and molecular physics, Atomic and molecular interactions with photons

## Abstract

We consider the model of two coupled oscillators with Kerr nonlinearities in the rotating-wave approximation. We demonstrate that for a certain set of parameters of the model, the multi-photon transitions occur between many pairs of the oscillator states simultaneously. Also, the position of the multi-photon resonances does not depend on the coupling strength between two oscillators. We prove rigorously that this is a consequence of a certain symmetry of the perturbation theory series for the model. In addition, we analyse the model in the quasi-classical limit by considering the dynamics of the pseudo-angular momentum. We identify the multi-photon transitions with the tunnelling transitions between the degenerate classical trajectories on the Bloch sphere.

## Introduction

For decades, the models consisting of interacting nonlinear oscillator modes attract much attention because of their importance for various fundamental concepts of quantum physics, quantum information and nonlinear dynamics. The phenomena present in such models include quantum chaos^1^, multi-stability^2^, dissipative phase transitions^3^, and dynamical tunnelling^4^. Nonlinear oscillator networks were suggested as a framework for universal quantum computation^5^, and also quantum nonlinear oscillators were suggested as a tool for non-classical states creation such as squeezed states^6^, entangled states^7,8^ and cat states^9^. Also, such models are a fundamental tool to study quantum-classical correspondence^10^.

The models of interacting nonlinear oscillators can be realised in various experimental setups including optomechanical systems^11^, trapped ions^12^ and superconducting circuits^13^. The presence of a dynamical bifurcation point was experimentally demonstrated in RF-driven Josephson junctions^14^ and transitions between two basins of attraction were observed in a nano-mechanical resonator. Also, non-degenerate parametric amplifiers were created based on the Josephson junction arrays^13^.

Lots of efforts are devoted to studying the nonlinear oscillator systems in the mesoscopic regime^3,10,15^. In this regime, it is possible to use the quasi-classical approximation, but the quantum effects are important as well. The interplay of complex classical dynamics and quantum effects such as quantum tunnelling leads to new interesting phenomena. For example, it was shown that in the model of a single oscillator mode with Kerr nonlinearity, tunnelling affects the switching rate between the stable states^16^.

The specifics of tunnelling in such systems are determined by the complex structure of the phase space. For the models exhibiting bi- or multi-stability, there exist several different classical trajectories with the same energies. This opens way for tunnelling transitions between the corresponding quantum states. However, as the tunnelling transition amplitudes are exponentially small, the certain resonant condition should be usually satisfied in order to achieve a non-vanishing tunnelling probability^4^. Also, the interconnection between tunnelling and multi-photon transitions was established in many systems^17–20^. In particular, for a single nonlinear oscillator with Kerr nonlinearity, tunnelling between different regions of the phase space is equivalent to simultaneous absorption or emission of many oscillator quanta in the quasi-classical limit.

In this work, we consider the model of two coupled nonlinear oscillators in the rotating-wave approximation (RWA)^21^. The classical limit of this model can be described as the dynamics of the pseudo-angular momentum on a two-dimensional sphere. Among the classical trajectories, there exist ones with equal energies, which makes tunnelling transitions between them possible. We show that tunnelling between such trajectories can be described with the perturbation theory in the coupling constant, and tunnelling transition is equivalent to the exchange by many quanta between the oscillators. We use high-order perturbation theory to study these processes and account for both resonant and non-resonant contributions. We identify the resonant condition for tunnelling between different classical trajectories and we prove rigorously that this resonant condition is satisfied simultaneously for many pairs of the oscillator states. Also, it is independent of the value of the coupling constant between oscillators, in contrast with many systems demonstrating similar behaviour (such as^22^). This fact is a consequence of an internal symmetry of the system Hamiltonian which we establish in all orders of the perturbation theory series. Also, we discuss how the presence of such symmetry modifies the energy spectrum and the structure of the eigenstates.

These results could have applications for quantum information processing and quantum state manipulation, in particular, the generation of the entangled states of two modes. We believe that the predicted features can be observed in systems with high-quality oscillator modes with a pronounced Kerr nonlinearity, such as the plasmon modes of Josephson junction arrays or the phonon modes of trapped ion ensembles. Both mentioned systems represent a natural realization of a system of coupled quantum nonlinear oscillators well isolated from the environment Also, the considered model is closely related to some models^23,24^ of dissipative time crystals (DTC)^25,26^. The results of this work could be useful to study the quantum effects in the DTC. In addition, the discovered symmetry extends the range of known symmetries in quantum–optical systems^27–30^.

## Multi-photon transitions between two coupled nonlinear oscillators

We consider the model of two oscillators with linear coupling and Kerr nonlinearities. Let the frequencies of the oscillators be $\omega _{1,2}$, and the Kerr shifts per oscillators quanta be $\alpha _{1,2}$. When the detuning between the oscillators $\Delta = \omega _2 - \omega _1$ is much smaller than the oscillators frequencies, one can neglect the counter-rotating terms in coupling (RWA)^21^, and the model Hamiltonian reads

1 $$\begin{aligned} \hat{H}=\omega _1 \hat{a}^{\dagger } \hat{a}+\omega _2\hat{b}^{\dagger } \hat{b} + \frac{\alpha _{1}}{2}\left( \hat{a}^{\dagger } \hat{a} \right) ^{2}+\frac{\alpha _{2}}{2} \left( \hat{b}^{\dagger } \hat{b} \right) ^{2}+ g\left( \hat{a}^{\dagger } \hat{b}+\hat{b}^{\dagger } \hat{a}\right) , \end{aligned}$$

where *g* is the coupling constant between the oscillators. As the counter-rotating terms are not present in (1), this Hamiltonian commutes with the total number of quanta operator $\hat{N} = \hat{a}^{\dagger } \hat{a} + \hat{b}^{\dagger } \hat{b}$ (we will denote its eigenvalues as *N*). Therefore, the total Hilbert space $\mathscr {H}$ of the considered model (1) splits into the direct sum of invariant Hilbert subspaces each corresponding to *N* quanta in the oscillators: $\mathscr {H} = \bigoplus \limits _{N=0}^{\infty } \mathscr {H}_N$, and also $\hat{H} = \sum _N \hat{H}_N$. The Hamiltonians $\hat{H}_N$ which act on the subspace $\mathscr {H}_N$ can be written with help of bra-ket notation as

2 $$\begin{aligned} \hat{H}_{N}= & {} \frac{1}{2}\left( \alpha _{1}+\alpha _{2}\right) \sum _{n=0}^{N} n(n - \mu _N)|n, N-n\rangle \langle n, N-n| \nonumber \\{} & {} +g \sum _{n=0}^{N} \sqrt{n(N-n+1)}\Big (|n-1, N-n+1\rangle \langle n, N-n|+ |n,N-n\rangle \langle n-1, N-n+1|\Big )+ \omega _2 N + \frac{\alpha _2 N^2}{2}, \end{aligned}$$

where $|n_a, n_b\rangle = \left| n_a\right\rangle \otimes \left| n_b\right\rangle$ are the oscillators Fock states ($n_a$ and $n_b$ are the numbers of quanta in modes *a* and *b* respectively), and

3 $$\begin{aligned} \mu _N = 2 \left( \Delta + \alpha _2 N \right) / \left( \alpha _1 + \alpha _2 \right) , \end{aligned}$$

**Figure 1:**
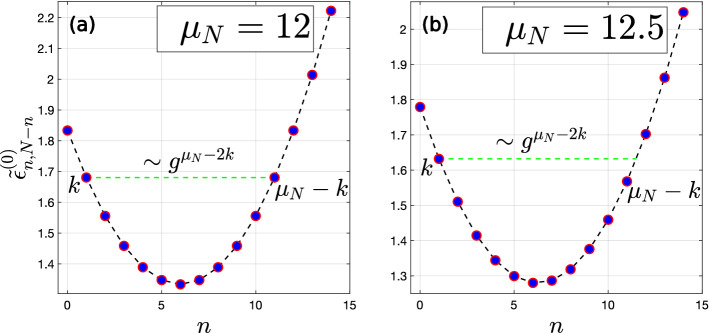
The dimensionless eigenenergies $\tilde{\varepsilon }^{(0)}_{n, N-n}=(\alpha _1 + \alpha _2) \varepsilon ^{(0)}_{n,N-n} / (\Delta + \alpha _2 N)^2$ (with $\varepsilon$ denoting the unnormalized eigenenergies) of the Hamiltonian (1) at $g=0$, $N=14$, $\alpha _2 / \alpha _1 = 1.1$, and (**a**) $\mu _N = 12$ and (**b**) $\mu _N = 12.5$ as functions of *n* for fixed *N*.

The model (1) is closely related to the model of a single Kerr oscillator^31^ in the classical external field. Namely, in the limit $N\rightarrow \infty$, $g\sqrt{N} = \text{const}$, the Hamiltonian $\hat{H}_N$ approaches the Hamiltonian of a single Kerr oscillator in the classical external field with nonlinearity $\alpha _1~+~\alpha _2$, detuning $\alpha \mu _N/2$, and the external field amplitude $~g\sqrt{N}$. This can be seen from Eq. (2) (Fig. 1).

In the following, we will focus on the case of relatively weak coupling. Moreover, in “The proof of the symmetry properties” sections and High-order perturbation theory for the degenerate energy levels, we will use the perturbation theory in *g* as a theoretical tool. Because of that, let us first consider the model without perturbation. In this case, the Hamiltonian (1) commutes both with $a^\dagger a$ and $b^\dagger b$, and its eigenstates are the Fock states $|n_a, n_b\rangle$ of the oscillators. The energy of each state $|n_a, n_b\rangle$ can be found easily from Eq. (1)

4 $$\begin{aligned} \varepsilon _{n_a,n_b}^{(0)} = \frac{\alpha _1}{2}n_a^2 + \frac{\alpha _2}{2}n_b^2 + \omega _1 n_a + \omega _2 n_b. \end{aligned}$$

The perturbation operator allows the oscillator to exchange quanta between each other. Namely, the perturbation directly couples $|n_a, n_b\rangle$ and $|n_a\pm 1, n_b\mp 1\rangle$. Because of that, the states $|n_a,n_b\rangle$ and $|n_a + k,n_a - k\rangle$ also become coupled in the order *k* by perturbation, and the transition amplitude between them is proportional to $g^{k}$. It turns out that at certain values of the detuning between the oscillators, such transitions (we will call them multi-photon) become resonant, and even at small couplings, the oscillators can exchange by *k* quanta simultaneously between each other.

This occurs because the unperturbed energies of the oscillators Fock states $|n,N-n\rangle$ in each subspace corresponding to *N* quanta have a parabolic dependence on *n*. The way how a sequence of the values $\varepsilon _{n,N}^{(0)}$ are arranged on a parabola depends on the value of the parameter $\mu _N$. One can easily see that at integer $\mu _N=m\in \mathbb {Z}$, many energies split into pairs with equal values: $\varepsilon ^{(0)}_{n,N-n} = \varepsilon ^{(0)}_{m-n,N-m+n}$ for $n = 0, \dots m$ Fig. 1(a). In contrast, for non-integer $\mu _N$ the unperturbed energies are non-degenerate Fig. 1(b). Also, for the case $\alpha _1 = \alpha _2$, the resonance condition is simultaneously satisfied for all *N*.

When $\mu _N$ is close to an integer, the degeneracy makes multi-photon transitions possible between the states $|n,N-n\rangle$ and $|m-n,~N-m+n\rangle$ when the perturbation is turned on. In other words, the integer values of $\mu _N$ at small *g* correspond to multi-photon resonances between $|n,N-n\rangle$ and $|m-n, N-m+n\rangle$.

**Figure 2:**
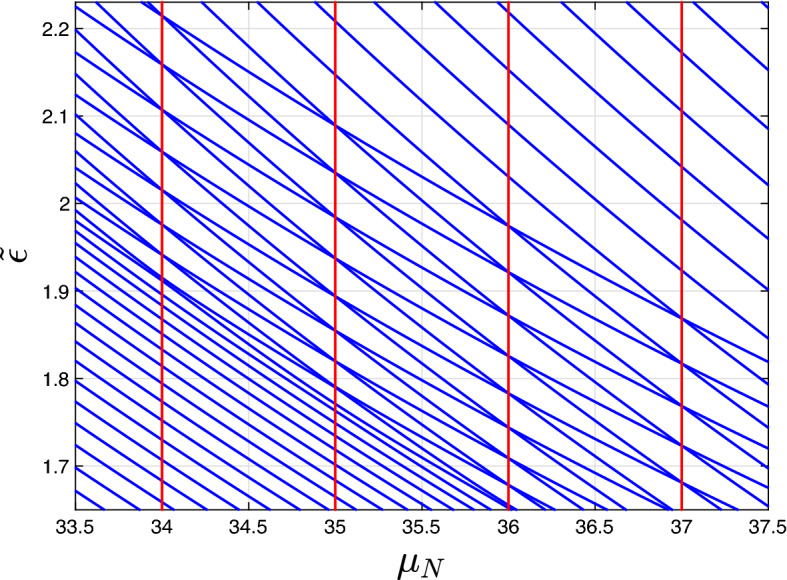
The dimensionless eigenenergies $\tilde{\varepsilon } = (\alpha _1 + \alpha _2) \varepsilon / (\Delta + \alpha _2 N)^2$ (with $\varepsilon$ denoting the unnormalized eigenenergies) of the Hamiltonian (1) corresponding to the subspace with $N = 46$ quanta at $\alpha _2 / \alpha _1 = 1.5$, $\Delta = \mu _N (\alpha _1 + \alpha _2)/2 - \alpha _2 N$, and $g = 0.32g_{\text{crit}}$ (see “Classical limit” section for the definition of $g_{\text{crit}}$) are shown as functions of $\mu _N$. All the anti-crossing points are located on the vertical lines corresponding to integer values of $\mu _N$.

However, the perturbation not only leads to multi-photon transitions between $|n, N - n\rangle$ and $|m - n, N - m + n\rangle$, but also induces the energy shifts of these levels due to the non-resonant coupling with other levels. In general, these shifts could lift the position of the multi-photon resonances from exactly $\mu _N \in \mathbb {Z}$, and to make them dependent on *g*. The surprising feature of the considered model is that these energy shifts are equal for every degenerate pair $|n, N - n\rangle$ and $|m - n, N - m + n\rangle$ (see Fig. 2). Because of that, simultaneous multi-photon resonances between many level pairs occur exactly at integer values of $\mu _N$ even at relatively large values of *g*.

In the absence of degeneracy in the unperturbed model (for the case of non-integer $\mu _N$), weak coupling between the oscillators leads only to a small *O*(*g*) perturbation of the Fock states of the system: the eigenstates remain close to Fock states. This is not the case when the multi-photon resonance condition is satisfied. Due to the reasons indicated above, even at small couplings the structure of the Hamiltonian eigenstates and the temporal dynamics of the system wave function for integer $\mu _N$ qualitatively differ from the case of non-interacting oscillators.

In this case, the leading-order contributions to the wave functions are symmetric and antisymmetric superpositions of the Fock states $|n,N-n\rangle$ and $|m-n, N-m+n\rangle$. For every $n = 0,\dots , m$,

5 $$\begin{aligned} |\psi _{n,m-n}^\pm \rangle \sim \frac{1}{\sqrt{2}} \big (|n,N-n\rangle \pm |m-n, N-m+n\rangle \big ) + O(g). \end{aligned}$$

**Figure 3:**
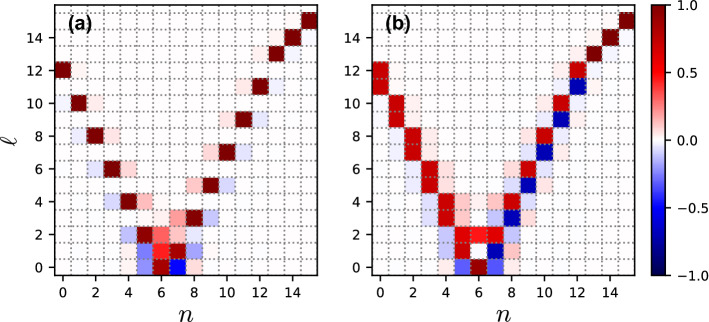
The expansion coefficients $c_{\ell , n}$ of the eigenstates of the Hamiltonian (1) at $N = 12$, $\alpha _2/\alpha _1 = 1$, $g/\alpha _1 \approx 0.03$, (**a**) $\Delta / \alpha _1 = 6.25$ and (**b**) $\Delta / \alpha _1 = 6.5$ are shown. For each pair $n, \ell$, a unit square centered at the corresponding point of the figure is drawn. The color of the square indicates the magnitude of $c_{n,\ell }$ according to the color bar.

This can be seen from the results of the numerical diagonalization of the Hamiltonian (1). We find the eigenstates of the Hamiltonian (1) in the Fock basis in the form $\left| \psi _{\ell }\right\rangle ~=~\sum _n c_{\ell , n}~\left| n, N-n\right\rangle$. The eigenstates are sorted in the ascending order according to their eigenenergies. Then, we plot the matrices of the expansion coefficients $c_{\ell , n}$ in Fig. 3 for the cases of integer (Fig. 3a) and non-integer $\mu _N$ (Fig. 3b). For each eigenstates shown in Fig. 3a, there is a single Fock state which has dominant contribution, whereas the eigenstates in Fig. 3b contain equal contributions from two Fock states as in Eq. (5).

**Figure 4:**
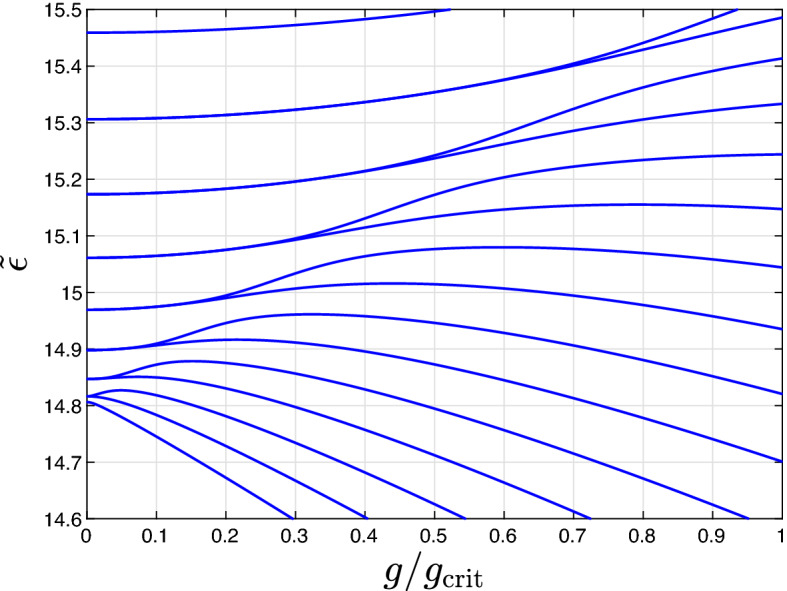
The dimensionless eigenenergies $\tilde{\varepsilon } = (\alpha _1 + \alpha _2) \varepsilon / (\Delta + \alpha _2 N)^2$ of the Hamiltonian (1) (with $\varepsilon$ denoting the eigenenergies) as functions of the coupling *g* for $\mu _N = 14$, $\alpha _2 / \alpha _1 = 1.5$, $N = 50$ and $\Delta = \mu _N (\alpha _1 + \alpha _2)/2 - \alpha _2 N$. For the definition of $g_\text {crit}$, see “Classical limit”.

The energy difference between the states $|\psi _{n,m-n}^\pm \rangle$ exhibiting the anti-crossing is determined by the multi-photon transition amplitude $\omega ^R_{n,m-n} \propto g^{m-2n}$ (see Fig. 4)

6 $$\begin{aligned} \varepsilon _{n,m-n}^+ - \varepsilon _{n,m-n}^- = 2\omega ^R_{n,m-n} + O\left( g^{m-2n+1}\right) . \end{aligned}$$

In addition, from Eqs. (5) and (6), for the initial state: $\left| \psi (0)\right\rangle ~=~\sum _{n} c_n \left| n, N-n\right\rangle$, one can find (at integer $\mu _N = m$) the following approximate solutions of the non-stationary Schrodinger equation

7 $$\begin{aligned} |\psi (t)\rangle = \sum \limits _{n}c_n e^{-\frac{i}{2}\left( \varepsilon _{n,m-n}^+ + \varepsilon _{n,m-n}^-\right) t} \Big [\cos {(\omega ^R_{n,m-n} t)} |n,N-n\rangle - i \sin {(\omega ^R_{n,m-n} t)} |m-n,N-m+n\rangle \Big ]. \end{aligned}$$

As can be seen, the system exhibits multi-photon Rabi oscillations between many pairs of the Fock states simultaneously.

**Figure 5:**
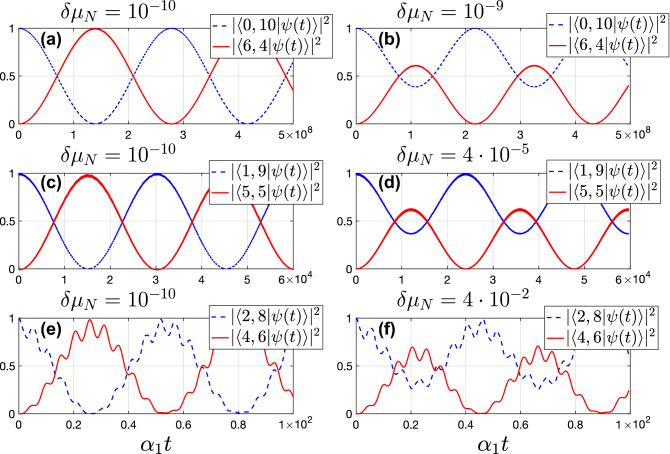
For the wave function $\left| \psi (t)\right\rangle$ solving the Schrodinger equation with the initial condition $\left| \psi (0)\right\rangle = \left| n,N-n\right\rangle$, the projections on $\left| n, N-n \right\rangle$ and $\left| m-n, N-m+n\right\rangle$ are shown for (**a**,**b**) $n = 0$, (**c**,**d**) $n = 1$, (**e**,**f**) $n = 2$, $N = 10$, $\alpha _2 / \alpha _1 = 1$, $\Delta = \mu _N (\alpha _1 + \alpha _2)/2 - \alpha _2 N \approx -4$, $g / \alpha _1 = 0.05$, $\mu _N = m + \delta \mu _N$, $m = 6$, and different $\delta \mu _N$.

In Fig. 5, we show the numerical solution of the Schrodinger equation for different Fock states taken as initial conditions. We plotted the squares of modulus of the overlappings between the wave function $\left| \psi (t)\right\rangle$ and the bra-states $\left\langle n,N-n\right|$, $\left\langle m-n,N-m+n\right|$ for different *n*. This result is in agreement with Eq. (7) for integer $\mu _N$. Also, for the case of non-integer $\mu _N = m + \delta \mu _N$ with sufficiently small $\delta \mu _N$, the amplitude of multi-photon Rabi oscillations between $|n, N-n\rangle$ and $|m-n, N-m+n\rangle$ decreases with increasing $\delta \mu _N$. They completely vanish when $(\alpha _1+\alpha _2)\delta \mu _N \gg \omega _{n,m-n}^R$ (see left and right panels of Fig. 5). In addition, there are corrections to Eq. (7) which come from the non-resonant contributions of the adjacent Fock states. They lead to the additional modulations of the Rabi oscillations with the amplitude $\sim g/\Delta$ and become more pronounced with increasing *n* due to the dependence of the transition matrix elements and the energy differences on *n*. They can be seen on the lower panels of Fig. 5. For other panels, they are also present but not resolved on the plots.

Also, let us briefly discuss the effect of weak dissipation on the considered multi-photon Rabi oscillations. In the presence of dissipation, each eigenstate of the system of coupled oscillators obtains a finite lifetime having the same order of magnitude as the decay rates $\gamma _a$, $\gamma _b$ of the individual bosonic modes^32^. The condition necessary to observe the Rabi oscillations between the states $|n,N-n\rangle$ and $|m-n,N-m+n\rangle$ is that the lifetime of the eigenstates $|\psi _{n,m-n}^\pm \rangle$ is much longer than the period of Rabi oscillations $\omega _{n,m-n}^R$, which leads to conditions $\gamma _{a,b} \ll \omega _{n,m-n}^R$. The requirements to observe the multi-photon transitions between two oscillator states become increasingly strict with the increasing order of the multi-photon transition. Thus, in the presence of dissipation, it is realistic to observe several transitions between the states $|n,N-n\rangle$ and $|m-n,N-m+n\rangle$ with the lowest values of $|m-2n|$.

The independence of the anti-crossings position on the value of *g* can have practical applications for the measurements of the Kerr coefficients of the oscillators. More precisely, assume the detuning $\Delta$ between the oscillators is scanned to measure some observable. Often, the multi-photon resonances lead to peaks/dips in the corresponding dependencies on $\Delta$. According to the above considerations, the positions of these peaks correspond to the integer multiples of the total nonlinearity independently of the coupling constant. This could be used for the measurement of $\alpha _1 + \alpha _2$.

In addition, the multi-photon transitions provide a way to prepare the entangled states of two oscillators. For example, according to Eq. (7), the initial Fock state $\left| n, N-n\right\rangle$ evolves into an entangled state at the proper choice of the interaction time (when $\omega ^R_{n,m-n} t \sim \pi / 4$).

## The proof of the symmetry properties

In this section, we provide a proof (on the physical level of rigour) of the presence of many simultaneous anti-crossings at $\mu _N \in \mathbb {Z}$ for the model (1).

Let us consider the coupling term between the oscillators as a perturbation. As commented above, at integer $\mu _N = m$, the unperturbed energies split into $\lfloor m/2 \rfloor$ pairs of degenerate levels. The perturbation leads to two effects: the shifts of the energy levels due to the non-resonant couplings, and the multi-photon transitions between the degenerate levels. The consistent analysis of both of the effects requires the usage of high-order perturbation theory.

Because of the degeneracies, one can not use directly the non-degenerate perturbation theory series in *g* for the system energies. However, at non-integer $\mu _N$ one can define the energies $\varepsilon _n(g)$ which correspond to the eigenstates evolving from the *n*-th Fock state $\left| n, N-n\right\rangle$ after the adiabatic switching of the perturbation. These energies (at non-integer $\mu _N$) can be found from non-degenerate perturbation theory as power series

8 $$\begin{aligned} \varepsilon _n(g) = \varepsilon _n^{(0)} + \sum \limits _{k = 1}^{\infty } \varepsilon _n^{(k)} g^k. \end{aligned}$$

(The expressions for the second and fourth order corrections could be found in Supplementary Information).

To some extent, this expansion is valid for $\mu _N \in \mathbb {Z}$ as well. Although at $\mu _N\in \mathbb {Z}$ the states $|n, N-n\rangle$ and $|m - n, N-m+n\rangle$ are degenerate, the perturbation couples them only in the order $|m-2n|$. Therefore, up to this order of the perturbation theory, they can be treated as non-degenerate ones. Also, the perturbation theory coefficients $\varepsilon _n^{(k)}$ are rational functions of *n* and $\mu _N$, therefore, they are well–behaved at $\mu _N\in \mathbb {Z}$. So, one can use the expansion (8) up to the order $|m-2n|$ in the degenerate case.

Surprisingly, the following identity is valid for the perturbation theory corrections at $\mu _N = m \in \mathbb {Z}$:

9 $$\begin{aligned} \varepsilon _n^{(k)} = \varepsilon _{\mu _N-n}^{(k)}. \end{aligned}$$

Therefore, the degeneracy between the levels $|n,N-n\rangle$ and $|m-n,N-n+m\rangle$ is not lifted in several lowest orders of the perturbation theory until the order $|m-2n|$. This explains the Eq. (6) and the results of the numerical diagonalization.

Strictly speaking, the identity (9) makes sense only for the case of the integer $\mu _N = m$. However, as the corrections $\varepsilon _{n}^{(k)}$ are rational functions of *n* and $\mu _N$, they can be analytically continued to the case of arbitrary real *n* and $\mu _N$. We will prove (9) for these analytical continuations.

The analytical continuation of $\varepsilon _{n}^{(k)}$ for the case of non-integer *n* can be made in a natural way for all *k*. For that, the whole energy spectrum $\varepsilon _{n}(g)$ should be continued to the case of non-integer *n*. To do this, let us assume that the Hamiltonian (1) acts on the space of all possible real $<<$numbers of quanta$>>$ $\nu$ with formally defined matrix elements as

10 $$\begin{aligned} \langle \nu , N - \nu -1| \hat{a} | \nu +1, N - \nu - 1\rangle = \left\langle \nu +1, N - \nu \left| \hat{a}^{\dagger }\right| \nu , N - \nu \right\rangle =\sqrt{\nu +1}, \end{aligned}$$

and analogously for $\hat{b}$ and $\hat{b}^\dag$. As one can see, in the case of an integer $\nu$, the definition of (10) reduces to the usual action of the bosonic creation and annihilation operators in the Fock space. We shall note that $\hat{a}$ and $\hat{a}^\dag$ are no longer Hermitian conjugate with each other for the case of non-integer $\nu$, so the Hamiltonian becomes non-Hermitian. However, one can check that the wave functions remain normalizable even for the Hamiltonian at the non-integer $\nu$.

The extended Hamiltonian acts invariantly on each of the subspaces $\mathscr {V}_\nu =\{|\sigma , N - \sigma \rangle : \sigma - \nu \in \mathbb {Z}\}$. (The subspace $\mathscr {V}_\nu$ consists of the ket vectors $|\sigma \rangle$ such as $\sigma - \nu \in \mathbb {Z}$.). Also, the subspaces of states $|\nu , N - \nu \rangle$ with negative integer $\nu$, $\nu \in [0, N]$ and integer $\nu > N$ are decoupled from each other. So, the extended Hamiltonian for all real $\nu$ acts exactly as the original Hamiltonian (1) on the subspace of $|n, N - n\rangle$, $n\in [0, N]$. We emphasize that the extension to the non-integer numbers of quanta should be taken as an auxiliary tool for the proof. Because, as we mentioned above, we are interested in the case of an integer $\nu$ which corresponds to the initial Hermitian Hamiltonian.

Until the end of this section, we will work in the subspace of a fixed number of quanta *N*. We will denote $\left| \nu , N - \nu \right\rangle$ as $\left| \nu \right\rangle$ for brevity.

Let us define the action of the extended Hamiltonian on $\mathscr {V}_\nu$ as $\mathscr {H}_{\nu , N}$. The operator $\mathscr {H}_{\nu , N}$ can be written in terms of the states $|\nu \rangle$ as

11 $$\begin{aligned} \hat{\mathscr {H}}_{\nu , N}=\frac{1}{2}\left( \alpha _{1}+\alpha _{2}\right) \sum \limits _{\sigma -\nu \in \mathbb {Z}} \sigma (\sigma -\mu _N)|\sigma \rangle \langle \sigma |+ g \sum \limits _{\sigma -\nu \in \mathbb {Z}} \sqrt{\sigma (N-\sigma +1)}(|\sigma -1\rangle \langle \sigma |+| \sigma \rangle \langle \sigma -1|). \end{aligned}$$

To prove the symmetry of the perturbation theory corrections to the energy, we will show the exact symmetry of the eigenstates of the extended Hamiltonian with respect to the replacement $\nu \rightarrow \mu _N - \nu$. Namely, we will prove that $\mathscr {H}_{\nu , N}$ and $\mathscr {H}_{\mu _N-\nu , N}$ have the same eigenenergies $\varepsilon _{\nu }(g)$ and $\varepsilon _{\mu _N - \nu }(g)$ respectively as a functions of *g*. For that, we will show that there exists a linear operator $\mathscr {T}$ such

12 $$\begin{aligned} \hat{\mathscr {H}}_{\nu , N}=\mathscr {T} I \hat{\mathscr {H}}_{\mu _N-\nu , N} I^{-1} \mathscr {T}^{-1}, \end{aligned}$$

where *I* is the isomorphism between the vector spaces spanned by vectors $\left| \nu \right\rangle$ and $\left| \mu _N - \nu \right\rangle$ respectively.

The existence of the operator $\mathscr {T}$ proves that the operators $\mathscr {H}_{\nu , N}$ and $\mathscr {H}_{\mu _N-\nu , N}$ have identical spectra at any *g*, which means that

13 $$\begin{aligned} \varepsilon _{\nu }(g) = \varepsilon _{\mu _N-{\nu }}(g), \qquad 2 \nu -\mu _N \notin \mathbb {Z}. \end{aligned}$$

In Supplementary Information, we explicitly construct the operator $\mathscr {T}$ and show that it can be expressed in the following form: $\mathscr {T} = UTV^{-1}$, where

14 $$\begin{aligned} \begin{aligned} T&= \left( \mathbbm {1} - \frac{2g}{\left( \alpha _{1}+\alpha _{2}\right) } \sum \limits _{\sigma - \nu \in \mathbb {Z}} \left| \sigma \right\rangle \left\langle \sigma + 1\right| \right) ^{- (N-\mu _N)}, \\ U&=\sum \limits _{\sigma } \sqrt{\frac{\Gamma (\sigma +1)}{\Gamma (N-\sigma +1)}}|\sigma \rangle \langle \sigma |, \quad V=\sum \limits _{\sigma } \sqrt{\frac{\Gamma \left( \mu _{N}-\sigma +1\right) }{\Gamma \left( N-\mu _{N}+\sigma +1\right) }}|\sigma \rangle \langle \sigma |. \end{aligned} \end{aligned}$$

After we proved the equality of the energies (13), let us turn back to the physically meaningful case of the integer $\nu$ and $\mu _N = m$. As we mentioned before, the energy $\varepsilon _{\nu }(g)$ can be decomposed in perturbation theory series of *g* (compare with Eq. (8)):

15 $$\begin{aligned} \varepsilon _{\nu }(g)=\frac{1}{2}(\alpha _1 + \alpha _2) \nu (\nu -\mu _N)+\sum _{k=1}^{\infty } \varepsilon _{\nu }^{(k)} g^{k}. \end{aligned}$$

From the equality (13) valid for all orders in *g*, one concludes that this holds for each term of the perturbation series:

16 $$\begin{aligned} \varepsilon _{\nu }^{(k)} = \varepsilon _{\mu _N-\nu }^{(k)} \quad \forall k, \quad 2 \nu -\mu _N \notin \mathbb {Z}. \end{aligned}$$

From Eq. (16), the equality (9) can be derived easily. The *k*-th order corrections $\varepsilon _{\nu }^{(k)}$ are rational functions of $\mu _N$ and $\nu$ and have no singularities at $2\nu - \mu _N \in \mathbb {Z}$ unless $k \geqslant 2|\mu _N-2\nu |$ (we prove it rigorously in the next sections). Therefore, the equality (16) holds even for the case of an integer $2\nu -\mu _N$, and also for $\nu , \mu _N \in \mathbb {Z}$. This concludes the proof of the equality (9).

We should note that the analogous properties hold for the model of a single nonlinear mode with Kerr nonlinearity driven by the classical external field (recovered as a limit of the considered model at $N \rightarrow \infty$). For this model, the equality of the perturbation theory corrections was checked^33^ for several lowest orders, and the sketch of the proof was given previously^34^.

## High-order perturbation theory for the degenerate energy levels

We have stated that non-degenerate perturbation theory corrections are symmetric with respect to replacement $n \rightarrow \mu _N - n$ in all orders. However, additional arguments are needed to relate this result to the physical case of $\mu _N \in \mathbb {Z}$. On one hand, due to the presence of degeneracy in the energy spectrum, one should use the degenerate perturbation theory. However, on the other hand, the off-diagonal matrix elements in the secular equation occur only in the $|m-2n|$-th order of perturbation theory. To demonstrate that until the $|m-2n|$-th order one can use the non-degenerate perturbation theory and to calculate the multi-photon amplitude via degenerate perturbation theory for higher orders than $|m-2n|$, it is convenient to apply Green’s function formalism. Namely, we consider the operator of Green’s function defined as

17 $$\begin{aligned} \hat{G}(\omega ) = \left[ \omega - \hat{H}\right] ^{-1}. \end{aligned}$$

If the spectrum of the problem $\sigma (\hat{H}) = \{\varepsilon _n, \left| \psi _n\right\rangle \}$ is known, Eq. (17) can be rewritten as follows

18 $$\begin{aligned} \hat{G}(\omega )=\sum _{n} \frac{\left| \psi _{n}\right\rangle \left\langle \psi _{n}\right| }{\omega -\varepsilon _{n}}. \end{aligned}$$

Thus, eigenenergies are poles of Green’s function and eigenfunctions could be calculated from residues of $\hat{G}$. The matrix element $G_{n,n}$ for each Fock state $|n\rangle$ can be calculated from the Dyson equation and reads

19 $$\begin{aligned} G_{n, n}(\omega )=\frac{1}{\omega -\varepsilon _{n}^{(0)}-\Sigma _{n}(\omega )}, \end{aligned}$$

where $\Sigma _n$ is the self-energy term, defined as the sum of all diagrams which start and finish at *n* and do not contain the Green’s function $G_{n,n}$. When $\Sigma _n$ is the regular function in the vicinity of $\omega = \varepsilon _n^{(0)}$, the position of the pole of $G_{n,n}$ can be found from the equation $G_{n, n}^{-1}(\omega )=0$ as power series in *g* which coincides with the result of usual non-degenerate perturbation theory expansion. However, if there exists another level with the same or close energy (this is $|m-n, N-m+n\rangle$ in our case), the self-energy itself acquires a pole in the vicinity of $\varepsilon _n^{(0)}$. The lowest-order perturbation theory term with a pole has the order $2|m-2n|$ in *g* because the corresponding diagram must contain at least one $G_{m-n,m-n}$ Green’s function. As the perturbation operator changes the number of quanta by one, at least $2|m-2n|$ perturbation vertices are required.

Because of the pole in the self-energy term in the vicinity of $\varepsilon _n^{(0)}$, the Green’s function poles positions cannot be found as simple perturbation series. For this case, it is convenient to consider the matrix Green’s function

20 $$\begin{aligned} \mathscr {G}=\left[ \begin{array}{ll} G_{n, n} &{} G_{n, m-n} \\ G_{m-n, n} &{} G_{m-n, m-n} \end{array}\right] . \end{aligned}$$

In terms of matrix Green’s function, Dyson equation could also be written with self-energy matrix $\Sigma$

21 $$\begin{aligned} \begin{aligned} \mathscr {G}&=\mathscr {G}^{(0)}+\mathscr {G}^{(0)} \Sigma \mathscr {G}, \\ \mathscr {G}^{(0)}&= \left[ \begin{array}{cc} \left( \omega - \varepsilon _n^{(0)}\right) ^{-1} &{} 0 \\ 0 &{} \left( \omega - \varepsilon _{m-n}^{(0)}\right) ^{-1} \end{array}\right] , \quad \Sigma =\left[ \begin{array}{cc} \Sigma _{n, n}(\omega ) &{} \Sigma _{n, m-n}(\omega ) \\ \Sigma _{m-n, n}(\omega ) &{} \Sigma _{m-n, m-n}(\omega ) \end{array}\right] . \end{aligned} \end{aligned}$$

The solution of Eq. (21) reads

22 $$\begin{aligned} \begin{aligned} \mathscr {G} = \left[ \left( \mathscr {G}^{(0)} \right) ^{-1} - \Sigma \right] ^{-1} = \begin{bmatrix} \omega - \varepsilon _n^{(0)} - \Sigma _{n,n}(\omega ) &{} -\Sigma _{n,m-n}(\omega ) \\ -\Sigma _{m-n,n}(\omega ) &{} \omega - \varepsilon _{m-n}^{(0)} - \Sigma _{m-n,m-n}(\omega ) \end{bmatrix}^{-1}. \end{aligned} \end{aligned}$$

The oscillator eigenenergies are the poles of the Green’s function. They could be found from the equation $\det \left( \mathscr {G}^{-1} \right) = 0$, and all their dependence on the perturbation is contained in the self-energy matrix $\Sigma$. The diagonal terms $\Sigma _{n,n}$ and $\Sigma _{m-n,m-n}$ correspond to non-resonant energy shifts and contain contributions of all even powers of *g*. In contrast, the off-diagonal term $\Sigma _{n,m-n}$ is proportional to $g^{m-2n}$ and is responsible for resonant multi-photon transition between the states $\left| n, N-n\right\rangle$ and $\left| m-n, N-m+n\right\rangle$. The leading term of its expansion in powers of *g* reads (see Supplementary Information)

23 $$\begin{aligned} \Sigma _{n, m-n}(\omega )=g^{m-2 n} \sqrt{\frac{(m-n) !}{n !} \frac{(N-n) !}{(N-(m-n)) !}} \frac{1}{\left( \omega -\varepsilon ^{(0)}_{n+1}\right) \ldots \left( \omega -\varepsilon ^{(0)}_{m-n-1}\right) }+O\left( g^{m-2 n + 1}\right) . \end{aligned}$$

Equations (21)–(23) explain the possibility to use the non-degenerate perturbation theory up to the $|m-2n|$-th order. According to Eq. (23), the off-diagonal terms in (22) (which correspond to the multi-photon resonance), have the order of $g^{m-2n}$. Therefore, they do not contribute to the *k*-th order of the perturbation theory when $k < |m-2n|$. For $k < |m-2n|$, the energies obtained from the secular equation will coincide with those obtained from the non-degenerate perturbation theory. To account for multi-photon transitions, one should consider the perturbation theory of order $k \geqslant |m-2n|$.

The term $\Sigma _{n, m-n}\left( \omega = \varepsilon _n^{(0)}\right)$ can be interpreted as the multi-photon transition amplitude between $\left| n, N-n\right\rangle$ and $\left| m-n, N-m+n\right\rangle$, and $\left| \Sigma _{n, m-n}\left( \varepsilon _n^{(0)}\right) \right|$ equals the frequency of the multi-photon Rabi transitions $\omega ^R_{n,m-n}$. Further, we will demonstrate that it can be treated as the tunneling amplitude in the quasiclassical limit.

## Classical limit

Multi-photon transitions described above with help of the formalism of the perturbation theory also have a quasi-classical interpretation as tunnelling transitions. For that, one should consider the classical limit of the studied model which is valid for the large number of quanta: $N \gg 1$, $\mu _N \gg 1$, $N/\mu _N = \text{const}$. To obtain the classical Hamiltonian of the system, one should replace the ladder operators in (1) with classical complex amplitudes^21,35^. This results in the following complex Hamilton function

24 $$\begin{aligned} H = \omega _1 |a|^2 + \omega _2 |b|^2 + \frac{\alpha _1}{2}|a|^4 + \frac{\alpha _2}{2} |b|^4 + g(a^*b + b^* a). \end{aligned}$$

Due to the conservation of the total number of quanta $N = |a|^2 + |b|^2$, the classical dynamics governed by this Hamiltonian can be described as the dynamics on the surface of the two-dimensional sphere. To show that, one should rewrite the classical Hamiltonian with help of the new pseudo angular momentum variables $L_x$, $L_y$, $L_z$ defined as

25 $$\begin{aligned} \begin{aligned} L_z&=\frac{1}{2}\left( |a|^2 - |b|^2\right) , \quad L_{+}=a^*b, \quad L_{-}= ab^*, \\ L_x&= \frac{1}{2}\left( L_{+} + L_{-}\right) , \quad L_y = \frac{1}{2i}\left( L_{+} - L_{-}\right) . \end{aligned} \end{aligned}$$

In terms of the components of $\textbf{L}$, the classical Hamiltonian (24) takes the form

26 $$\begin{aligned} H = -\Delta \left( L_z + \frac{N}{2}\right) + \frac{\alpha _1}{2}\left( L_z + \frac{N}{2} \right) ^2 + \frac{\alpha _2}{2}\left( L_z - \frac{N}{2} \right) ^2 + 2gL_x + \omega _2 N. \end{aligned}$$

**Figure 6:**
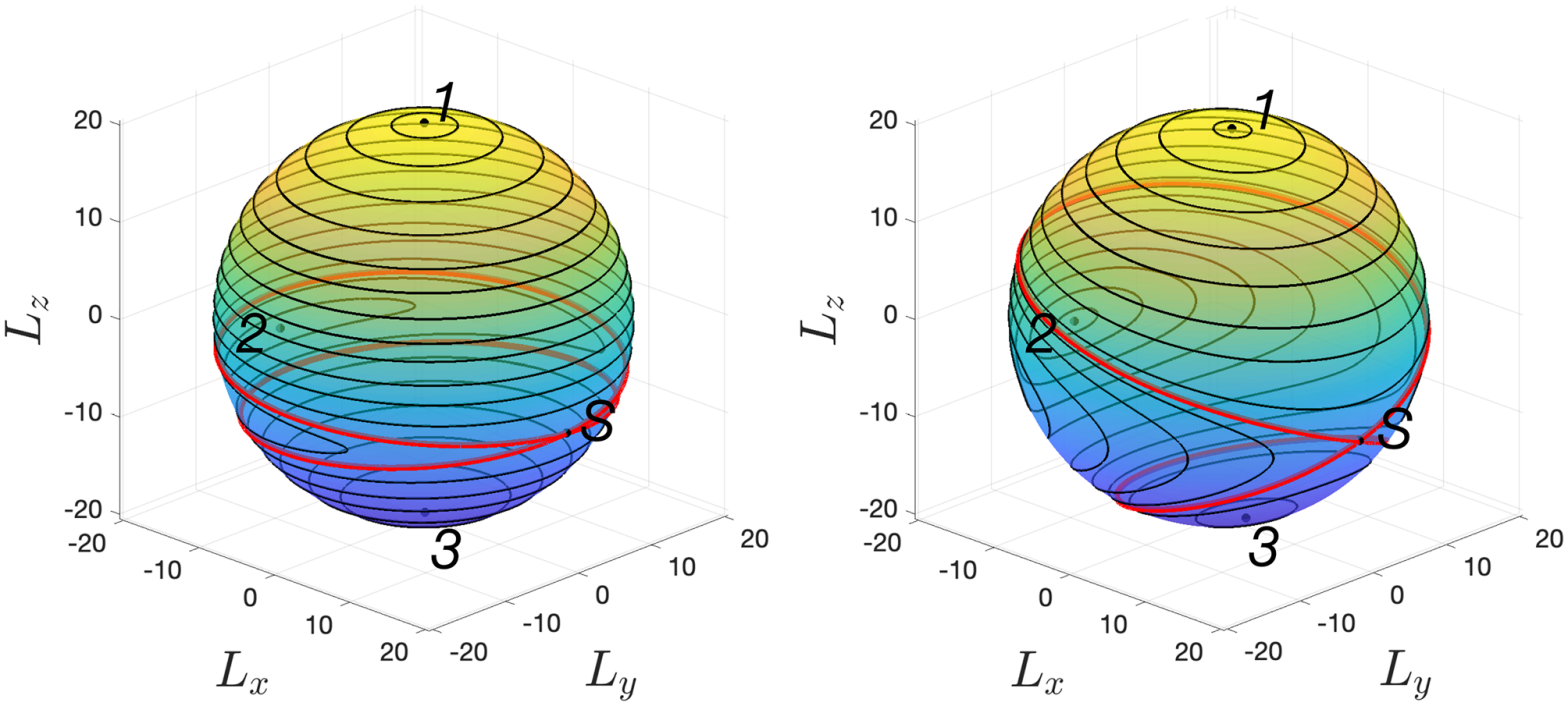
Classical trajectories on the surface of Bloch sphere. Black dots correspond to equilibrium points (three stable—1, 2, 3, and one unstable—*S*). The red curve is a Separatrix. Here the parameters are as follows: $N=40$, $\alpha _2/\alpha _1 = 0.5$, $\Delta /\alpha _1 = 0.25$, $\mu _N = 27$, $g/\alpha _1 \approx 0.1211$ (left), and $g/\alpha _1 \approx 1.816$ (right). This corresponds to the dimensionless coupling strength: $\sqrt{\beta } \approx 0.0103$ (left), and $\sqrt{\beta } \approx 0.1544$ (right).

**Figure 7:**
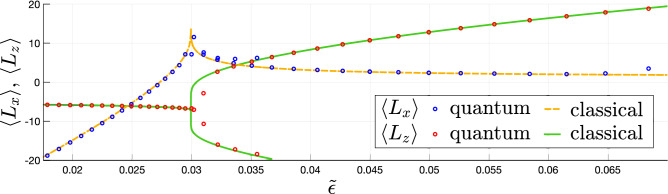
The average values of the pseudo angular momenta components $L_x$ and $L_z$ over the classical trajectories with the dimensionless energy $\tilde{\varepsilon }$ (orange dashed and green solid line) and over the eigenstates of the quantum Hamiltonian with the eigenenergies $\tilde{\varepsilon }$ (red and blue circles). The averages are calculated at the following values of the Hamiltonian parameters: $N=40$, $\alpha _2/\alpha _1 = 0.5$, $\Delta /\alpha _1 = 0.25$, $\mu _N = 27$, and $g/\alpha _1 \approx 1.816$.

The conservation of the total angular momentum $L^{2}=L_{x}^{2}+L_{y}^{2}+L_{z}^{2} = N(N+2)/4$ (which is equivalent to the conservation of the total number of quanta in the oscillators) allows describing the classical dynamics with help of the classical phase portrait on the Bloch sphere (see Fig. 6): the trajectories in the $L_{i}$ space are defined by the conservation of the number of quanta and the Hamilton function (26). Due to the quantum-classical correspondence, the eigenstates of the quantum Hamiltonian (1) correspond to a discrete set of classical trajectories on the Bloch sphere. We demonstrate this by comparing the period-averaged values of the classical momenta $L_x$, $L_z$ with the quantum-mechanical averages over the eigenstates of (1) for non-integer $\mu _N$ in Fig. 7 (the average of $L_y$ equals zero). As one can see from Fig. 7, the averages calculated from the classical model are close to the quantum averages everywhere except the vicinity of the separatrix.

Let us discuss the structure of the phase portrait in detail. At $g = 0$, the Hamiltonian is a function of $L_z$ only, therefore, the classical trajectories are the circles in the $L_x$, $L_y$ plane. At non-zero *g*, the trajectories are no longer concentric circles. Also, a self-intersecting trajectory (separatrix) emerges which divides the phase portrait into three regions with a stable point inside each one (denoted as $<<1>>$, $<<2>>$ and $<<3>>$). At a certain critical value of $g = g_\text{crit}$, a saddle-node bifurcation occurs, and one of the stable point merges with the unstable stationary state $<<S>>$. At larger *g*, only two stable points remain. Depending on the value of the ratio $N/\mu _N$, the unstable point $<<S>>$ can merge with the point $<<1>>$ or with the point $<<3>>$. If $N/\mu _N > 1$, the point $<<3>>$ merges with the point $<<S>>$, otherwise, the point $<<1>>$ merges with point $<<S>>$.

The value of $g_\text{crit}$ can be found by analysing the positions of the extrema of the Hamiltonian (26) as a function of $L_i$. After casting the coupling constant to a dimensionless form $\beta = {8g^2N}/{(\alpha _1 + \alpha _2)^2\mu _N^3}$ (the dimensionless coupling strength), we obtained the following expression for $\beta _\text{crit}$ (see derivation in Supplementary Information):

27 $$\begin{aligned} \beta _\text{crit} = \frac{\gamma }{2}\left( \gamma ^{\,2/3} - (\gamma -1)^{ 2/3}\right) ^3, \end{aligned}$$

where $\gamma = N/\mu _N$. Also, in the limit of $\gamma \rightarrow \infty$, one recovers the result for the model of a single Kerr oscillator with classical driving: at $\gamma \rightarrow \infty$, $\beta _\text {crit} \rightarrow 4/27$^36,37^.

**Figure 8:**
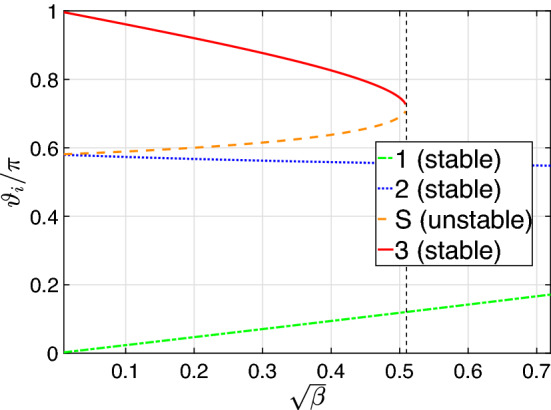
The angles $\vartheta _i = \pi /2 - \arctan (L_z^{(i)}/\big |L_x^{(i)}\big |)$ as a function of $\sqrt{\beta }$ for the *i*-th equilibrium point, $i \in \{1,2,3,S \}$. Black dashed line corresponds to $\beta = \beta _\text {crit}$. The points numbers correspond to the ones in Fig. 6. Here the parameters are as follows: $N=40$, $\alpha _2 / \alpha _1 = 0.5$, $\Delta / \alpha _1 = 2.5$, $\mu _N = 30$, and $g = \sqrt{\beta (\Delta +\alpha _2 N)^3/N(\alpha _1 + \alpha _2)}$.

The dependence of the positions of the stable states on the dimensionless coupling strength $\beta$ is presented in Fig. 8. For each of the equilibrium points $i \in \{1,2,3,S \}$, the value of the polar angle $\vartheta _i = \pi /2 - \arctan (L_z^{(i)}/\big |L_x^{(i)}\big |)$ is plotted (see Supplementary Information for more details). At $\beta = \beta _\text {crit}$ (vertical black dotted line), the states $<<S>>$ (unstable) and $<<3>>$ (stable) merge. Also, both of the angles $\vartheta _1$ and $\vartheta _2$ approach $\pi /2$ at $\beta \gg \beta _\text {crit}$, which corresponds to the diametrically opposite stationary points in the *X*-*Y* plane.

Now let us discuss how tunnelling modifies the classical picture and establish the relation between tunnelling and multi-photon transitions. Tunnelling transitions are possible providing that there exist classical trajectories with the same energies. Because of the structure of the classical phase portrait, this holds for $g < g_\text{crit}$. In this case, tunnelling transitions are possible between the classical trajectories from regions 1 and 3. At small *g*, the classical trajectories are close to the circles in the *X*-*Y* plane (see Fig. 6, left panel) and can be identified with the oscillators Fock states: the Fock state $|n,N-n\rangle$ corresponds to the circle with $L^2 = N^2/4$, $L_z = N/2 - n$. For this case, one can directly apply the results of “Multi-photon transitions between two coupled nonlinear oscillators” and “The proof of the symmetry properties” sections and deduce that at the integer $\mu _N$, resonant tunnelling transitions are possible between the trajectories with $L_z = N/2 - n$ and $L_z = N/2 - \mu _N + n$. For larger coupling values, the eigenstates are no longer the Fock states, and the classical trajectories are no longer circles in the *X*-*Y* plane. However, we state that the condition for resonant tunnelling remains the same for all values of $g \in [0, g_\text{crit}]$. This is supported by the fact that the calculation of “The proof of the symmetry properties” section is performed in all orders of the perturbation theory. Because of that, it fully takes into account the modification of the classical trajectories at larger *g*. In addition, the numerical diagonalization of the Hamiltonian (1) demonstrates that the behaviour shown in Fig. 2 (simultaneous anticrossings at the integer $\mu _N$) persists for the whole range $g \in [0, g_\text{crit}]$.

## Conclusions

For the model of two coupled quantum oscillators with Kerr nonlinearities and linear coupling, we studied the multi-photon transitions between the oscillators. We showed that for certain parameters of the model, the resonant condition for multi-photon transitions is simultaneously satisfied for many pairs of the oscillator Fock states. This holds even for the moderate coupling strength between oscillators, and this is the consequence of a special symmetry of the oscillators Hamiltonian.

The latter is related to the structure of the perturbation series of the model eigenenergies and was proven with help of analytical continuation of the Hamiltonian for non-integer numbers of quanta.

Also, in the quasi-classical limit, the phase space of the two coupled oscillators can be mapped on a sphere, and the multi-photon transitions can be interpreted as tunnelling transitions between the trajectories lying in different regions of the classical phase space. Thus, when the resonant condition is satisfied, tunnel transitions affect the whole region of the classical phase space.

We believe that the results obtained in this work could be relevant for the experiments involving high-quality oscillator modes with low occupation numbers, such as the plasmon modes of the Josephson junction arrays or phonon modes of trapped ions ensembles. In particular, the independence of the multi-photon resonances positions could be used for the measurements of the Kerr coefficients of the modes. Also, the multi-photon transitions provide a way to create the entangled states of two oscillators. In addition, the obtained results could be used for certain models of dissipative time crystals in the quantum regime, and the symmetry discovered in the considered model may allow obtaining new exact results in quantum-optical systems.

## Supplementary Information

Supplementary Information.

## Data Availability

The datasets used and/or analysed during the current study available from the corresponding author on reasonable request.

## References

[1] Adamyan HH, Manvelyan SB, Kryuchkyan GY (2001). Chaos in a double driven dissipative nonlinear oscillator. Phys. Rev. E.

[2] Tadokoro Y, Tanaka H, Dykman MI (2018). Driven nonlinear nanomechanical resonators as digital signal detectors. Sci. Rep..

[3] Zhang XHH, Baranger HU (2021). Driven-dissipative phase transition in a Kerr oscillator: From semiclassical $$\cal{PT}$$ symmetry to quantum fluctuations. Phys. Rev. A.

[4] Serban I, Wilhelm FK (2007). Dynamical tunneling in macroscopic systems. Phys. Rev. Lett..

[5] Goto H (2016). Universal quantum computation with a nonlinear oscillator network. Phys. Rev. A.

[6] Maslova NS, Mantsevich VN, Arseyev PI, Sokolov IM (2019). Tunneling current induced squeezing of the single-molecule vibrational mode. Phys. Rev. B.

[7] Joshi C, Jonson M, Andersson E, Öhberg P (2011). Quantum entanglement of an harmonic oscillators. J. Phys. B Atom. Mol. Opt. Phys..

[8] Teh RY (2020). Dynamics of transient cat states in degenerate parametric oscillation with and without nonlinear Kerr interactions. Phys. Rev. A.

[9] Dodonov V, Malkin I, Man’ko V (1974). Even and odd coherent states and excitations of a singular oscillator. Physica.

[10] Andersen CK (2020). Quantum versus classical switching dynamics of driven dissipative Kerr resonators. Phys. Rev. Appl..

[11] Pistolesi F (2018). Bistability of a slow mechanical oscillator coupled to a laser-driven two-level system. Phys. Rev. A.

[12] Ding S, Maslennikov G, Hablützel R, Loh H, Matsukevich D (2017). Quantum parametric oscillator with trapped ions. Phys. Rev. Lett..

[13] Muppalla PR (2018). Bistability in a mesoscopic Josephson junction array resonator. Phys. Rev. B.

[14] Siddiqi I (2005). Direct observation of dynamical bifurcation between two driven oscillation states of a Josephson junction. Phys. Rev. Lett..

[15] Shirai T, Todo S, de Raedt H, Miyashita S (2018). Optical bistability in a low-photon-density regime. Phys. Rev. A.

[16] Maslova NS, Anikin EV, Mantsevich VN, Gippius NA, Sokolov IM (2019). Quantum tunneling effect on switching rates of bistable driven system. Laser Phys. Lett..

[17] Wallraff A, Duty T, Lukashenko A, Ustinov AV (2003). Multiphoton transitions between energy levels in a current-biased Josephson tunnel junction. Phys. Rev. Lett..

[18] Dykman MI, Fistul MV (2005). Multiphoton antiresonance. Phys. Rev. B.

[19] Maslova NS, Anikin EV, Gippius NA, Sokolov IM (2019). Effects of tunneling and multiphoton transitions on squeezed-state generation in bistable driven systems. Phys. Rev. A.

[20] Wang R (2019). Identification of tunneling and multiphoton ionization in intermediate Keldysh parameter regime. Opt. Express.

[21] Carmichael HJ (1998). Statistical Methods in Quantum Optics 1.

[22] Owerre S, Paranjape M (2015). Macroscopic quantum tunneling and quantum–classical phase transitions of the escape rate in large spin systems. Phys. Rep..

[23] Seibold K, Rota R, Savona V (2020). Dissipative time crystal in an asymmetric nonlinear photonic dimer. Phys. Rev. A.

[24] Lledó C, Szymańska MH (2020). A dissipative time crystal with or without $$\mathbb{Z} _2$$ symmetry breaking. New J. Phys..

[25] Sacha K, Zakrzewski J (2017). Time crystals: A review. Rep. Prog. Phys..

[26] Else DV, Monroe C, Nayak C, Yao NY (2020). Discrete time crystals. Ann. Rev. Condens. Matter Phys..

[27] Lamata L (2019). Symmetry in quantum optics models. Symmetry.

[28] Mangazeev VV, Batchelor MT, Bazhanov VV (2021). The hidden symmetry of the asymmetric quantum Rabi model. J. Phys. A Math. Theor..

[29] Li Z-M, Batchelor MT (2021). Hidden symmetry and tunneling dynamics in asymmetric quantum Rabi models. Phys. Rev. A.

[30] Lu X, Li Z-M, Mangazeev VV, Batchelor MT (2021). Hidden symmetry in the biased Dicke model. J. Phys. A Math. Theor..

[31] Drummond PD, Walls DF (1980). Quantum theory of optical bistability. 1. Nonlinear polarisability model. J. Phys. A Math. Gen..

[32] Breuer H-P, Petruccione F (2007). The Theory of Open Quantum Systems.

[33] Risken H, Vogel K (1988). Quantum tunneling rates in dispersive optical bistability for low cavity damping. Phys. Rev. A.

[34] Anikin EV, Maslova NS, Gippius NA, Sokolov IM (2019). Enhanced excitation of a driven bistable system induced by spectrum degeneracy. Phys. Rev. A.

[35] Dodonov VV, Man’ko VI (2003). Theory of Nonclassical States of Light.

[36] Vogel K, Risken H (1990). Dispersive optical bistability for large photon numbers and low cavity damping. Phys. Rev. A.

[37] Maslova NS, Johne R, Gippius NA (2007). Role of fluctuations in nonlinear dynamics of a driven polariton system in semiconductor microcavities. JETP Lett..

