# Supplementary material for: The symmetry in the model of two coupled Kerr oscillators leads to simultaneous multi-photon transitions
Source: Sci Rep. 2023 Feb 21;13:2997. doi: 10.1038/s41598-023-30197-8 (PMC9944305; doi:10.1038/s41598-023-30197-8)
Supplement: Supplementary file 1 — Supplementary Information. [file 41598_2023_30197_MOESM1_ESM.pdf]

Supplementary Information: The symmetry in the model of two coupled Kerr oscillators leads to simultaneous multi-photon transitions

Bogdan Y. Nikitchuk[1,2*], Evgeny V. Anikin[1,3], Natalya S. Maslova[4], and Nikolay A. Gippius[1]

[1]Skolkovo Institute of Science and Technology, 121205 Moscow, Russia
[2]Moscow Institute of Physics and Technology, Dolgoprudny, 141701 Moscow Region, Russia
[3]Russian Quantum Center, Moscow 121205, Russia
[4]Quantum Technology Centrum, Department of Physics, Lomonosov Moscow State University, 119991 Moscow, Russia
[*]nikitchuk.bia@phystech.edu

ABSTRACT

Here we put Supplementary information to the main text

1 Non-degenerate perturbation theory corrections for the energy levels

In this section, we present the explicit expressions for the second- and the fourth-order non-degenerate perturbation theory corrections to the oscillator energy levels. The second-order perturbation correction for the level n reads

$$g^2\varepsilon_n^{(2)} = \frac{|V_{n,n-1}|^2}{\varepsilon_n^{(0)} - \varepsilon_{n-1}^{(0)}} + \frac{|V_{n,n+1}|^2}{\varepsilon_n^{(0)} - \varepsilon_{n+1}^{(0)}}, \tag{1}$$

where

$$V_{k,n} = g\left(\sqrt{n+1}\sqrt{N-n}\,\delta_{k,n+1} + \sqrt{n}\sqrt{N-n+1}\,\delta_{k,n-1}\right). \tag{2}$$

For the two levels n and $m-n$, the expressions (1) are not explicitly symmetric. However, the contributions of the levels $n-1$ and $n+1$ have different signs and partially cancel each other. By algebraic manipulations, one can obtain

$$\varepsilon_n^{(2)} = \frac{1}{\alpha_1+\alpha_2}\frac{(2n-\mu_N)^2 - \mu_N^2 + 2N(\mu_N+1)}{(2n-\mu_N)^2-1}, \tag{3}$$

which turns out to be symmetric with respect to the replacement $n \to \mu_N - n$. Much lengthier calculations also lead to the symmetric expression for the fourth-order correction

$$\begin{aligned}
\varepsilon_n^{(4)} &= \frac{1}{(\alpha_1+\alpha_2)^3}\frac{N^2A+NB+C}{\left((2n-\mu_N)^2-1\right)^3\left((2n-\mu_N)^2-4\right)},\\
A &= -12(2n-\mu_N)^4 + 4(2n-\mu_N)^2\left(5\mu_N^2+10\mu_N+11\right) + 4\left(7\mu_N^2+14\mu_N+4\right),\\
B &= 12(2n-\mu_N)^4(\mu_N-1) - 4(2n-\mu_N)^2\left(5\mu_N^3+5\mu_N^2+\mu_N-11\right) - 4\left(7\mu_N^3+7\mu_N^2-10\mu_N-4\right),\\
C &= (2n-\mu_N)^6 - 3(2n-\mu_N)^4\left(2\mu_N^2+3\right) + (2n-\mu_N)^2\left(5\mu_N^4+2\mu_N^2+20\right) + 7\mu_N^4 - 20\mu_N^2.
\end{aligned} \tag{4}$$

2 The symmetry of the generalised Hamiltonian

In this section, we prove the identity defined by Eq. (12), $\hat{\mathscr{H}}_{\nu,N} = \mathscr{T} I \hat{\mathscr{H}}_{\mu_N-\nu,N} I^{-1}\mathscr{T}^{-1}$ which proves the equivalence of two instances of the generalised oscillator Hamiltonians for the indices ν and $\mu_N-\nu$ (see Eqs. (11) and (14)).

First of all, one can check that after the action of the isomorphism I, the right-hand-side of Eq. (12) reads

$$I\hat{\mathscr{H}}_{\mu_N-\nu,N}I^{-1} = \frac{1}{2}(\alpha_1+\alpha_2)\sum_{\sigma-\nu\in\mathbb{Z}}\sigma(\sigma-\mu_N)\left|\sigma\right\rangle\left\langle\sigma\right| + g\sum_{\sigma-\nu\in\mathbb{Z}}\sqrt{(\mu_N-\sigma)(N-\mu_N+\sigma+1)}(\left|\sigma+1\right\rangle\left\langle\sigma\right|+\left|\sigma\right\rangle\left\langle\sigma+1\right|). \quad (5)$$

Second of all, let us define the new operators

$$\hat{H}_1 = U^{-1}\mathscr{H}_{\nu,N}U, \quad \hat{H}_2 = V^{-1}I\mathscr{H}_{\mu_N-\nu,N}I^{-1}V. \quad (6)$$

Using expressions (14) for U and V, it easy to obtain that these operators can be written as

$$\hat{H}_1 = \frac{1}{2}(\alpha_1+\alpha_2)\sum_{\sigma-\nu\in\mathbb{Z}}\sigma(\sigma-\mu_N)\left|\sigma\right\rangle\left\langle\sigma\right| + g\sum_{\sigma-\nu\in\mathbb{Z}}(\sigma(N-\sigma+1)\left|\sigma-1\right\rangle\left\langle\sigma\right|+\left|\sigma\right\rangle\left\langle\sigma-1\right|), \quad (7)$$

$$\hat{H}_2 = \frac{1}{2}(\alpha_1+\alpha_2)\sum_{\sigma-\nu\in\mathbb{Z}}\sigma(\sigma-\mu_N)\left|\sigma\right\rangle\left\langle\sigma\right| + g\sum_{\sigma-\nu\in\mathbb{Z}}((\mu_N-\sigma+1)(N-\mu_N+\sigma)\left|\sigma-1\right\rangle\left\langle\sigma\right|+\left|\sigma\right\rangle\left\langle\sigma-1\right|). \quad (8)$$

In terms of these operators, the identity of Eq. (12) takes the form

$$\hat{H}_1T = T\hat{H}_2, \quad (9)$$

Let us note that all the operators in Eq. (9) depend on g.

For $g=0$, it is obvious that $\hat{H}_1=\hat{H}_2$, and $T=\mathbb{1}$. For non-zero g, let us search for the operator T obeying Eq. (9) in the form of the Taylor series in g:

$$T = \mathbb{1} + \frac{2g}{(\alpha_1+\alpha_2)}T^{(1)} + \left(\frac{2g}{(\alpha_1+\alpha_2)}\right)^2 T^{(2)} + \dots. \quad (10)$$

After substituting the expression (10) for T into Eq. (9), one gets the identities for the matrix elements $T_{\sigma,\sigma'}$ of the Taylor expansion coefficients of T:

$$\sigma(\sigma-\mu_N)T^{(k+1)}_{\sigma,\sigma'} + (\sigma+1)(N-\sigma)T^{(k)}_{\sigma+1,\sigma'} + T^{(k)}_{\sigma-1,\sigma'} = \sigma'(\sigma'-\mu_N)T^{(k+1)}_{\sigma,\sigma'} + (\mu_N-\sigma'+1)(N-\mu_N+\sigma')T^{(k)}_{\sigma,\sigma'-1} + T^{(k)}_{\sigma,\sigma'+1}. \quad (11)$$

It may be easily proven by induction that the only non-zero matrix elements of the Taylor coefficients are $T^{(k)}_{\sigma,\sigma+k}$, and the values

$$T^{(k)}_{\sigma,\sigma+k} = \frac{(N-\mu_N)\dots(N-\mu_N+k-1)}{k!} = \frac{(N-\mu_N+k-1)!}{k!(N-\mu_N-1)!}, \quad (12)$$

indeed obey the identity (11). Also, let us note that $T^{(k)}_{\sigma,\sigma+k}$ coincide with the Taylor coefficients of the function

$$(1-x)^{-(N-\mu_N)} = \sum_{k=0}^{\infty}\binom{N-\mu_N+k-1}{k}x^k, \quad |x|<1. \quad (13)$$

So, the transformation operator T can be cast to a more compact form

$$T = \left(\mathbb{1} - \frac{2g}{(\alpha_1+\alpha_2)}\sum_{\sigma-\nu\in\mathbb{Z}}\left|\sigma\right\rangle\left\langle\sigma+1\right|\right)^{-(N-\mu_N)}. \quad (14)$$

3 Energy splitting due to multi-photon resonance

In this section, we calculate the matrix Green's function to demonstrate that the degeneracy in the spectrum of the model lifts at higher orders of perturbation theory. First of all, let us write the following matrix, containing the matrix element of the full Green's function

$$\mathscr{G} = \begin{bmatrix} G_{n,n} & G_{n,m-n} \\ G_{m-n,n} & G_{m-n,m-n} \end{bmatrix}, \tag{15}$$

here $G_{i,j} = \langle i|\hat{G}|j\rangle$ — matrix element of the Green's function. The matrix function (15) satisfy the following Dyson equation

$$\mathscr{G} = \mathscr{G}^{(0)} + \mathscr{G}^{(0)}\Sigma\mathscr{G}. \tag{16}$$

This Dyson equation can be written in the form of the diagram, see Fig. 1.

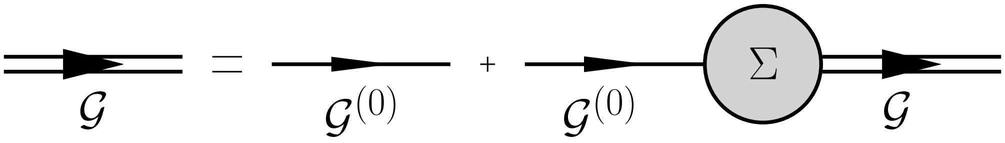

Figure 1. Feynman diagram for the Dyson equation for the matrix Green's function $\mathscr{G}$.

In the equation (16) and in Fig. 1, we introduced the notation

$$\mathscr{G}^{(0)} = \begin{bmatrix} \left(\omega - \varepsilon_n^{(0)}\right)^{-1} & 0 \\ 0 & \left(\omega - \varepsilon_{m-n}^{(0)}\right)^{-1} \end{bmatrix}, \quad \Sigma = \begin{bmatrix} \Sigma_{n,n}(\omega) & \Sigma_{n,m-n}(\omega) \\ \Sigma_{m-n,n}(\omega) & \Sigma_{m-n,m-n}(\omega) \end{bmatrix}. \tag{17}$$

In the formula (17), $\Sigma_{i,j}(\omega)$ it is a sum of all diagrams that start at i, finish at j and do not contain the indices i and j in between. In other words, $\Sigma_{i,j}(\omega)$ has the following form

$$\Sigma_{i,j}(\omega) = \sum_{k_1} \frac{V_{i,k_1}V_{k_1,j}}{\omega - \varepsilon_{k_1}^{(0)}} + \sum_{k_1,k_2} \frac{V_{i,k_1}V_{k_1,k_2}V_{k_2,j}}{(\omega - \varepsilon_{k_1}^{(0)})(\omega - \varepsilon_{k_2}^{(0)})} + \ldots + \sum_{k_1,\ldots,k_\ell} \frac{V_{i,k_1}V_{k_1,k_2}\ldots V_{k_\ell,j}}{(\omega - \varepsilon_{k_1}^{(0)})\ldots(\omega - \varepsilon_{k_\ell}^{(0)})} + \ldots. \tag{18}$$

Due to the fact of Hermiticity of the considered Hamiltonian, irreducible terms are conjugate: $\Sigma_{n,m-n}(\omega) = \Sigma^*_{m-n,n}(\omega)$ (in our case they are real). One can write the formal solution of Dyson Eq. (16)

$$\mathscr{G} = \left[\left(\mathscr{G}^{(0)}\right)^{-1} - \Sigma\right]^{-1} = \begin{bmatrix} \omega - \varepsilon_n^{(0)} - \Sigma_{n,n}(\omega) & -\Sigma_{n,m-n}(\omega) \\ -\Sigma_{m-n,n}(\omega) & \omega - \varepsilon_{m-n}^{(0)} - \Sigma_{m-n,m-n}(\omega) \end{bmatrix}^{-1}. \tag{19}$$

Poles of (19) are given by the following transcendental equation

$$\left(\omega - \varepsilon_n^{(0)} - \Sigma_{n,n}(\omega)\right)\left(\omega - \varepsilon_{m-n}^{(0)} - \Sigma_{m-n,m-n}(\omega)\right) = |\Sigma_{n,m-n}(\omega)|^2. \tag{20}$$

To analyse the irreducible term (18), let us look at the matrix element of the perturbation part

$$V_{k,n} = g\left(\sqrt{n+1}\sqrt{N-n}\,\delta_{k,n+1} + \sqrt{n}\sqrt{N-n+1}\,\delta_{k,n-1}\right). \tag{21}$$

It is clear from (21) that non-vanishing terms satisfy $k = n \pm 1$.

Using (20), one can calculate the leading-order contribution to the non-diagonal part of the self-energy matrix:

$$\Sigma_{n,m-n}(\omega) = \frac{V_{n,n+1}\ldots V_{m-n-1,m-n}}{(\omega - \varepsilon_{n+1}^{(0)})\ldots(\omega - \varepsilon_{m-n-1}^{(0)})} \propto g^{m-2n}. \tag{22}$$

Now let us prove that the splitting between two eigenenergies at the integer values of μ_N indeed occurs only in the order $|m-2n|$ of g. For that, we will use the symmetry of the perturbation theory corrections discussed above (in Section 2 of the supplementary), and in Section 2 of the main text.

The eigenenergies can be obtained from Eq. (20). This equation can be solved with help of the perturbation theory expansions of $\Sigma_{i,j}(\omega)$.

One can see that the right part is equal to zero up to the $|m-2n|$-th order of g. So, let us first consider the equations

$$\begin{aligned} \omega - \varepsilon_n^{(0)} - \Sigma_{n,n}(\omega) = 0, \\ \omega - \varepsilon_{m-n}^{(0)} - \Sigma_{m-n,m-n}(\omega) = 0, \end{aligned} \tag{23}$$

separately. Let us denote the roots of these equations as $\tilde{\varepsilon}_1(\mu_N)$ and $\tilde{\varepsilon}_2(\mu_N)$ respectively. As $\Sigma_{i,j}(\omega)$ are regular in the vicinity of integer μ_N, $\tilde{\varepsilon}_1(\mu_N)$ and $\tilde{\varepsilon}_2(\mu_N)$ are also regular and can be decomposed in power series in g.

We are interested in the degenerate or almost-degenerate case: in this case, $\tilde{\varepsilon}_1(\mu_N)$ and $\tilde{\varepsilon}_2(\mu_N)$ are very close to each other. It is convenient to consider the value of $\mu_N = \mu_N^*$ such as $\tilde{\varepsilon}_1(\mu_N^*) = \tilde{\varepsilon}_2(\mu_N^*)$. For $g = 0$, $\mu_N^* = m$.

Let us now solve (20) for the values of μ_N which are close to μ_N^*. For these values, each expression in (23) can be replaced by $\omega - \tilde{\varepsilon}_i$, $i = \{1,2\}$. Also, the argument of $\Sigma_{n,m-n}(\omega)$ can be replaced by $(\tilde{\varepsilon}_1 + \tilde{\varepsilon}_2)/2$ in the leading order of g. After that, the transcendental equation (20) becomes quadratic, and its roots $\varepsilon_{n,m-n}^{\pm}$ can be easily found

$$\varepsilon_{n,m-n}^{\pm} \approx \frac{\tilde{\varepsilon}_1 + \tilde{\varepsilon}_2}{2} \pm \sqrt{\left(\frac{\tilde{\varepsilon}_1 - \tilde{\varepsilon}_2}{2}\right)^2 + |\Sigma_{n,m-n}|^2}, \tag{24}$$

The energy spliting reads

$$\varepsilon_{n,m-n}^{+} - \varepsilon_{n,m-n}^{-} \approx 2\sqrt{\left(\frac{\tilde{\varepsilon}_1 - \tilde{\varepsilon}_2}{2}\right)^2 + |\Sigma_{n,m-n}|^2}. \tag{25}$$

The Eqs. (24) and (25) give the general leading-order expressions for the energies near the levels anti-crossing caused by multi-photon transition. According to Eq. (25), the minimal level splitting is achieved at $\mu_N = \mu_N^*$.

Now we will apply these results for the considered model of two coupled nonlinear oscillators. Let us recall that this model has the symmetry of the non-degenerate perturbation theory corrections. This symmetry allows to show that the energy splitting at $\mu_N \in \mathbb{Z}$ remains of order $\sim g^{m-2n}$ even though in general case μ_N^* depends on g.

Now let us write $\tilde{\varepsilon}_1$ and $\tilde{\varepsilon}_2$ in the form of power series in g

$$\begin{aligned} \tilde{\varepsilon}_1(\mu_N) = \varepsilon_n^{(0)} + c_2 g^2 + c_4 g^4 + \ldots, \\ \tilde{\varepsilon}_2(\mu_N) = \varepsilon_{m-n}^{(0)} + d_2 g^2 + d_4 g^4 + \ldots. \end{aligned} \tag{26}$$

Although $\tilde{\varepsilon}_i(\mu_N)$ do not coincide exactly with the eigenenergies of the oscillator, their perturbation series coincide with the non-degenerate perturbation series for $\varepsilon_n(g)$ and $\varepsilon_{m-n}(g)$ up to the order $2|m-2n|$. For $\tilde{\varepsilon}_1(\mu_N)$, this can be verified by comparing the diagrammatic expansions for $\Sigma_{n,n}(\omega)$ and $\Sigma_n(\omega)$ (see Section 3). These expansion coincide up to the order $2|m-2n|$. Therefore, the same is valid for $\tilde{\varepsilon}_1$ and ε_n, as the latter is the solution of the equation $\omega - \varepsilon_n^{(0)} - \Sigma_n(\omega) = 0$.

Therefore, in Eq. (26) for the case $\mu_N \in \mathbb{Z}$, all the coefficients c_k and d_k are equal to the non-degenerate perturbation theory corrections $c_2 = d_2 = \varepsilon_n^{(2)}$, $c_4 = d_4 = \varepsilon_n^{(4)}$, etc up to the order $2|m-2n|$. As a result, at $\mu_N = m \in \mathbb{Z}$, $\tilde{\varepsilon}_1(\mu_N) - \tilde{\varepsilon}_2(\mu_N) \sim g^{2(m-2n)}$. Therefore, at the integer values of μ_N,

$$\varepsilon_{n,m-n}^{+} - \varepsilon_{n,m-n}^{-} = 2\left|\Sigma_{n,m-n}\left(\varepsilon_n^{(0)}\right)\right| + O\left(g^{m-2n+1}\right) \approx 2\omega_{n,m-n}^{R}, \tag{27}$$

where $2\omega_{n,m-n}^{R}$ is the Rabi frequency of the multi-photon transition between the states $|n, N-n\rangle$ and $|m-n, N-m+n\rangle$. This means that the positions of anti-crossings indeed remain unshifted as stated in Sections 1 and 3. More rigorously, one can prove that $\mu_N^*(g) = m + O\left(g^{2(m-2n)+1}\right)$.

By substituting the actual values of the perturbation matrix elements and the energies into the expression (22), one can find

$$\omega_{n,m-n}^{R} = \frac{1}{2}(\alpha_1 + \alpha_2)\left(\frac{2g}{\alpha_1 + \alpha_2}\right)^{m-2n} \sqrt{\frac{(m-n)!}{n!} \frac{(N-n)!}{(N-(m-n))!}} \frac{1}{(m-2n-1)!^2}. \tag{28}$$

4 The equilibrium points of the phase portrait and the critical coupling strength

In this section, we find the critical value of the dimensionless coupling strength β corresponding to the bifurcation on the phase portrait of two coupled oscillators. For that, we find the positions of the equilibrium points on the phase space from the equations of motion for L_i and analyse their dependence on g. From the Hamiltonian in terms of pseudo angular momentum operators (26), the equations of motion for the angular momenta can be found with help of the Poisson brackets of L_i: $\{L_i, L_j\} = \varepsilon_{ijk} L_k$ (which follow from the quantum–mechanical commutators of $\hat{L}_i$). The resulting equations of motion take the form

$$\begin{cases} \frac{dL_x}{dt} = -(\alpha_1+\alpha_2)L_yL_z - \frac{1}{2}\left(\alpha_1 N - \alpha_2 N - 2\Delta\right)L_y, \\ \frac{dL_y}{dt} = (\alpha_1+\alpha_2)L_xL_z + \frac{1}{2}\left(\alpha_1 N - \alpha_2 N - 2\Delta\right)L_x - 2gL_z, \\ \frac{dL_z}{dt} = 2gL_y. \end{cases} \tag{29}$$

Since we are looking for equilibrium points, all derivatives should be equal to zero. For all the equilibrium points, $L_y = 0$, therefore, they can be found from the equation $dL_y/dt = 0$ together with the total pseudo momentum conservation law:

$$\begin{cases} (\alpha_1+\alpha_2)L_xL_z + \frac{1}{2}\left(\alpha_1 N - \alpha_2 N - 2\Delta\right)L_x - 2gL_z = 0, \\ L_x^2 + L_z^2 = \frac{N(N+2)}{4} \approx (N/2)^2, \quad N \gg 1. \end{cases} \tag{30}$$

The equilibrium points can be conveniently found with help of the angular parametrisation for L_x and L_z: $2L_z = N\cos\vartheta$, $2L_x = N\sin\vartheta$. The Eq. (30) reduce to the following trigonometric equation for ϑ:

$$\sin\vartheta + \left(1 - \frac{\mu_N}{N}\right)\tan\vartheta - \frac{4g}{N(\alpha_1+\alpha_2)} = 0, \quad N \gg 1. \tag{31}$$

At the critical value of g, two roots of this equation merge. However, a more convenient way to find it is not to explicitly examine the dependence of the roots of (31) on g but to consider formally the coupling constant g as a function of ϑ. The merging points of the roots correspond to the extrema of $g(\vartheta)$. By performing this procedure, one can obtain the following expression for the dimensionless coupling strength $\beta = 8g^2N/(\alpha_1+\alpha_2)^2\mu_N^3$:

$$\beta_{\text{crit}} = \frac{\gamma}{2}\left(\gamma^{2/3} - (\gamma-1)^{2/3}\right)^3, \tag{32}$$

where $\gamma = N/\mu_N$.
